# The Medical Pilgrim's Progress

**Published:** 1858-01

**Authors:** Benjamin W. Richardson

**Affiliations:** Physician to the Royal Infirmary for Diseases of the Chest; and Lecturer on Physiology and Public Hygiene at the School.


### ORIGINAL COMMUNICATIONS.

### THE MEDICAL PILGRIM'S PROGRESS:

BEING THE INTRODUCTORY LECTURE DELIVERED AT THE GROSVENOR PLACE SCHOOL OF MEDICINE, OCT. 1, 1857.

By BENJAMIN W. RICHARDSON, M.D., L.R.C.P.;
Physician to the Royal Infirmary for Diseases of the Chest; and Lecturer on Physiology and Public Hygiene at the School.

Gentlemen,—My colleagues have done me the honour to ask me to speak the first word, on the opening of this medical session. I accept the task with much pleasure and some anxiety; for, while it is difficult to invent new ideas or new topics for the subject matter of an introductory discourse, it is still more difficult to repeat what has been so often spoken before, with so much of eloquence, force, good taste, and feeling.

I asked the colleague who moved into my acceptance this pleasing duty, what I should say to you? His reply was indicative of the practical surgeon—"Give them something that they can see." Well! I have cast about for some subject of the kind suggested by my friend; but, as I see nothing myself of the illustrative type befitting the occasion, and demonstrable to the visual sense, it were vain for me to call your attention to this experimental wonder, or that surgical weapon. I must therefore be content to deal simply in thoughts and suggestions, in hopes and outshadowings; and you must be content to see while you hear. A vision of words is before you.

But who are before me? A students' assembly? No—yes. I hope a students' assembly always, and I the youngest of all. A student need not be a man; but a man, to be a man, must be a student. *Ergo*, we are all students. But some are students only,—others are students who have passed their pupilage. With the latter I shall have nothing to do this day, unless they choose to accept the student's simple position. The pure student, the elementary student, is my man now; the rest are hearers. I shall therefore address myself to my audience in elementary language, speaking boldly what I think, but simply, that the youngest may understand.

These preliminaries settled, and a proper understanding made, you await the theme. What is to come?

By rule and plummet, as I am advised, it would be a right thing to begin with a word about this very school; a word, of course, in strong commendation of this school, and of your discerning faculties; and all that—Society for the Promotion of Mutual Applause. On this topic I must, for the sake of good taste and feeling, be brief and honest; and so I say candidly that, while every student who enters this school can learn his profession if he will, so he can also learn it in other schools if he will, since the will is the royal way. Further, I would add that it is the student who makes the school, not the school the student. Whichever one of you, educated in these walls in past times, now, or in future, shall make for yourselves an honest reputation, and for the world a benefactor, in so far shall such an one make this place renowned and its teachers celebrated.

You expect me next, possibly, to say a great deal about the profession which you have chosen; and this of course in strong commendation of the profession and of your discerning choice, and all that—Society for the Promotion of Mutual Applause again—Limited Liability this time. The Society fails once more. I am not going to admit that, by comparison, medicine is second to any profession; though this negation says but little for physic. I am not going to preach to you the weak points of our science; for these you will learn soon enough for all practical purposes; but I am going rather to try to show to you who are to be the after-day representatives of medicine, on what the future greatness of medicine must be based—what you must live for, and hope for, and die for, if you would set the seal of merit on the work of this day.

THE MEDICAL PILGRIM'S PROGRESS—That is my theme.

And, as I stand here holding a trust which is as solemn as it is honourable, so is it my business to discharge that trust with true honesty of purpose as of word. There must be no misunderstanding between us. It must be assumed that you are anxious that the result of your pilgrimage shall end in success; and I must endeavour to set you forth fairly on your journey; so that, knowing the worst, you may be ever striving after the best.

You have doubtless all come to this new work with grand anticipations and resolutions. Rattling up to this modern Babylon, your enthusiasm has been raised to its full pitch. The train came not fast enough, while your hearts kept rapid harmony with your thoughts. The enthusiasm is healthy, and must not be checked, but directed rather into its right course; and this not for your own personal interests merely, but for the interest and honour of medicine as a profession.

Know then, that in expending your enthusiasm in the medical journey there are two paths that you may follow—the one broad, easy, and delusive—the other narrow, difficult, and uninviting, but the only true.

In the broad and easy path there are already numerous wanderers; for, as I say, the prospect is as tempting as it is false. The wanderers are in groups or classes. Let us inspect a few of them.

First, there is the great class of men of means. These can give *fêtes* to their fellows, and trust to their "governors" in all emergencies: moreover they can fight against time, and wait until something turns up to their advantage. These men, born with the celebrated silver spoon in their mouths, arrive sometimes at envied positions, which, by the way, they rarely sustain. Taking them all in all, they are not to be envied, when the best is said for them. They are the spoiled children of fortune, and are generally dissatisfied with themselves and everybody. Born not to work, they work *per* force, despising the labour of their own hands, however meritorious it may be. Born to the luxury of idleness, they are ignorant of the luxury of industry. Born to exercise from their cradle a petty rule, they grow up captious, supercilious, insolent. They form, in a word, the class which I shall designate the *Young Ladies in Physic.* Ready made vinegar, as Chemist Punch teaches, may always be obtained by praising one of them to another; and, so long as they remain unticketed amongst the extinct animals, Miss Blackwell, M.D., need, I take it, make no further proselytes.

There is another class in the broad highway; the men of this group aim at creeping towards distinction, not by their industry, but by the ignoble project of recognising the weakest of all human traits—the vanity of their fellows and their superiors, and by paying insincere deference to that vanity. These take to heart the modern poet's axiom,

> "That bowing down, and bowing down,

> Is the way to get on in London town."

They act on this motto, and too often they find it answer. Now observe, that in marking out these men, I am not insinuating a thought against the honest admiration of great and good men; but I am speaking against any attempt at progress through false adulation of the little great; and against that class of persons, whom I brand as *the Toadies in Physic*, to distinguish them from all others, and from the *young ladies* in particular.

Once more, and not to go further into detail, there is on the broad and easy way, a group of strolling idlers of a tawdry and mean caste; the drowsy, helpless, care-for-nothings, ne'er-do-wells. These men take things as they come, and let them go as they go. "They muddle away their incomes in paying their debts." The sun rises and sets on them; but what to them is the sun, except as a sort of necessary, and sometimes unpleasant flunkey? Often dissolute from the first, they become mere non-entities at last; they drawl on, deriding a progress they cannot follow, and die as if they had not been born. Why they were ever born is a dead secret; but born they are in great numbers, and, in my language, they furnish the class called the *Old Women in Physic,* which Miss Blackwell, M.D., very much and very properly despises.

You, none of you, wish to rank either with the *young ladies,* the *toadies,* or the *old women,* do you? Not a bit of it. Then let us leave them altogether in disgust, and pass from their highway to the difficult but manly route.

The narrow but difficult path in medicine is rendered sacred by the foot-prints which have indented it. Here trod Hippocrates. Here held he converse with the air, and the clouds, and the floods; with man in death, with man in birth. Here he drew up and transcribed those tablets which we retain with so much pride and admiration, and read to-day as though they were written but yesterday. Here Vesalius, with stout heart and unwearied step, moved on, climbing even the gibbet for the wherewithal for study, and stealing anatomy, and making it a science amidst the clamours of a stupid, ignorant, and superstitious community. In this path walked William Harvey, avoiding no ascent, however difficult, and gaining one at last which threw open to his vision, a new world. Here that child of the chisel, John Hunter, strove, and by unfailing energy proved, almost for the first time in the history of intellect, that genius may be a mere child of industry. Here, in a sentence, has every man who may be nominated a master in physic striven; and here, too, must you strive.

Does any one feel that the course thus marked out is too hard for common men; that genius is required for it, Herculean power, more strength than such an one possesses? The feeling is good; but blend with it no misgivings, for, as many a coward grows bold in the wars, so the weak, because untutored mind grows stronger and stronger as the labour of intellect grows more severe.

We often have to listen to a great deal of talk about genius; about the force of genius, the fire of genius, and such like phrases: as though genius were a principle *sui generis;* a kind of mental malformation dependent on a lobule of brain-stuff seated in some special recess in particular noddles. There is nothing of the kind; but there is certainly in some men a happy combination of ordinary mental faculties, which gives to these men a natural aptitude for logical and bold reasoning, and which sets them above their fellows. Such effective combination is made up of three very simple elements, which we will call *force, fearlessness*, and *fact*. Whoever has these, has the genius for science; and although education alone will never positively supply these elements, yet, as they are more or less inherent in every man, so are they brought out and strengthened by every educational exercise.

Remember then this triplet of faculties, and encourage their natural development in yourselves. Encourage *force* by care, by industry, by knowledge. Encourage *fearlessness* by determining that neither self-love, nor the risk of work, shall prevent you from accomplishing any honourable and possible task. Encourage *fact* as the keystone of your force, and your fearlessness; and you will be the representatives of genius beyond expectation.

But remember this also, that genius will not show itself by your constantly thinking about it, or by your fostering the notion that you are the sepulchres, or very mines of the great hidden treasure. *The Genius Angel in these days sets no kisses on the lips of babes in long clothes.* No, no. It is the faculty of true genius to know nothing of its own life. "What particular quality of mind led you to your victories?" was a question once asked of him whose statue is not a stone's throw hence "Common sense," was the immediate and honest reply. The hero of a hundred fights laid no claim to the special faculty. His successes rested on the three principles already named—force, fearlessness, and fact; combined, these constituted his common sense; and on them his fame rests after him.

Grasping then manfully the pilgrim's staff, and setting your feet in the right way, you must inure and qualify for your journey. You must prepare yourselves physically; you must prepare yourselves mentally. I mean by this, that you must learn so to govern the body as to bring it into healthy concord with the mind; for mind is in a great measure based on body. It is not my intention to read you a sermon on morals; for I accept you all as gentlemen in the full sense of the term, and a true gentleman is a man of morals: therefore I speak not of morals, but of physical training; of the development of muscle, and bone, and lungs, and heart, and brain; since your mind can only attain its full power, or retain it, while these physical forces are in working order.

Not to be wearisome, I will give you one general precept, which is the key to a thousand more. *Carry simplicity of life to the boundary of abstemiousness.*

As an architect can, if he will, build this house of stone, that of wood, and the other of brick; so can a man, by what he takes in, build up his ever-changing house of a body as he will, or nearly so. And as he builds, so in a great measure does he extend or narrow the power of his intellect. If he overbuild, or imprudently build, his mind pays the forfeit. Imbecility is the first-born child of luxury. I need not stay to prove this fact, it is so evident. In the history of men as individuals, as families, as nations, it is the one prominent truth. On the other side, the history of men as individuals, as families, as nations, proves with equal force the truth, that strength and vigour of intellect are inseparably connected with simplicity of the corporeal life.

We talk largely about the blessings of luxuries and creature comforts. But what positions do these bear in their influence on the world when compared with their opposites? They are idiots only who connect force with material wealth. On the contrary, there is not a single intellectual triumph, at all god-like in its magnitude, that has not arisen out of the so called *poverty* of this little earth; from the souls of men who, reared not in the lowest phases of deprivation, but far enough away from luxury, grew up having the powerful mind, blended with the simply nourished but healthy physical body.

I beg you, therefore, as you would advance rapidly, to make simplicity of life your first point. Brush up your recollections of the men whom you would most wish to imitate, and let their habits and practices guide yours.

You have heard, doubtless, of a certain great American patriot, named Benjamin Franklin, the man who first captured the lightning flash, and suggested the wire conductor which runs down your village spire; a man of genius this Franklin,—a mere working printer in his early days, and an immortal printer in those latter days when he became the political star and the glory of his country. There is a characteristic anecdote related of Franklin, which will bear repetition. When young, he established the *Pennsylvania Gazette,* and soon afterwards found occasion to remark with some degree of freedom on the public conduct of one or two persons of high standing in Philadelphia. This course was disapproved of by some of his patrons, who sought an opportunity to convey to him their views on the subject, and what they represented to be the opinion of his friends. He listened patiently, and replied by requesting that they would favour him with their company at supper, and bring with them the other gentlemen who had expressed dissatisfaction. The time arrived, and the guests assembled. He received them cordially, and listened again to their friendly reproofs of his editorial conduct. At length supper was announced; but when the guests had seated themselves around the table, they were surprised to see nothing before them but two puddings made of coarse meal, called "sawdust puddings" in the common phrase, and a stone pitcher filled with water. He helped them all, and then applied himself to his own plate, partaking freely of the repast, and urging his friends to the same. They taxed their politeness to the utmost, but all in vain; their appetites refused obedience to the will. Perceiving their difficulty, Franklin at last arose and said:—"My friends, any one who can subsist upon sawdust puddings and water, as I can, needs no man's patronage."

As Physiologist, it will be my business some day to show you that this anecdote must be taken only in its comparative meaning, and that sawdust puddings are not fitted for the perpetual food even of philosophers; but the moral of the story is not less striking; and it is the moral that I wish to imprint on your minds. And the moral is this; that a man of simple manner and life can do anything in reason, while a man of luxurious life can only prosper by riding on the shoulders of his fellows.

At the same time, bear always in mind, that frugality is in no way necessarily connected either with meanness, selfishness, or pettiness. The luxurious world may misinterpret you on this point; but mind not the world: it is your debtor, and you are not selfish because you attune your souls to the work before you. Make sure of that.

The great Morgagni, father of pathology, and master in physic, was personally abstemious to an extreme, his biographers say to a fault; and criticise the man for his self-sacrifice. Poor biographers! Poor fools! Why do such critics presume to write? Where would have been the astounding labours of our Morgagni, had he not instinctively fashioned his body to his mind? The work could not have been done in two lifetimes, by nerve more pampered. His industry gave birth to his genius, and his simplicity of manner fostered his industry.

Connected with the physical training of the body, I recommend to you strongly the continued and systematic strengthening of the muscular power. Our profession, lending itself too readily to the Old Wife's stereotype of a doctor, makes a grand and universal mistake in this respect. A doctor may pipe a sentimental song in an elegant carbonic acid depôt, called a drawing room. Yes; that is allowable, although a hearty laugh, which is the most healthy exercise in nature, is forbidden by prim society. The doctor may play a rubber at whist, with a whining old maid for a partner; that is excusable, unavoidable sometimes. He may mildly go through a dance; but he must not romp or polka; certainly not. He may drink wine after dinner till his head swims; that is essential. He may, in fact, do everything of this kind that is effeminate and enervating; but Heaven protect the man if he ventures to play tennis, cricket, or any other game that shall call into unwonted play his elegant, half-useless, mute-at-a-funeral-clad limbs.

Gentlemen, without encouraging you to any injurious pursuits, I call upon you as the coming representatives of medicine, to disobey the old woman oracle, which dictates these absurd conventialities. Innocent, active pleasures are the salt of life; and the more vigorous the man, the more vigorous the mind. I take it that much of the namby-pambyism, vapidity, and anæmic conceit, which too much characterize all the learned professions, are due to this slow progress of effemination, this undermining of the soul. Go to the British Association for the Advancement of Science—that parliament of intellect! and observe there who are the Science-Hercules. You will find them amongst the men who are most genial, best body-built, least conventional.

As another means of strengthening the physical organism, and of securing therewith the development of mental power, I urge on you strongly, as an amusement, the practical study of mechanical laws. Every lesson here learned is a lesson in practical mathematics; and the manufacture of a mere wheelbarrow by an educated man is worth a dozen problems of Euclid. In addition, the Esculapian has, throughout the whole of his career, to study and treat the most beautifully constructed of all machines; so that the more he knows of practical mechanics, the more he knows of man.

And yet another valuable process arises out of the practice of physical exercise; I mean promptitude in action; readiness, without the feeling of drudgery, to do at once, and to the best advantage, every work that has to be done. "Procrastination is the thief of time"—and nothing so surely produces procrastination as inertia of body. Mental labours in the development of their full force require inevitably some amount of physical industry; and you will find, if ever you come to the exercise of the purest labours of mind, that the preparatory step to each section of work is to throw off an indescribable sensation of physical idleness. My belief indeed is, that the mind is never idle in a strict sense; and that, although brain has to do all the thinking, it is muscle that has to do all the wearying. Physiologically, indeed, this is explained. For, as every art, physical or mental, is a tax levied upon the respiration and circulation; and as the functions of respiration and circulation are functions dependent on the energy of muscle, so is muscle a prime mover of mind, and so must it the more carefully be kept in full and vigorous force. The men who measure intellect by mere size of head and brain, comprehend but half the physical problem. A finely balanced brain is unquestionably a grand acquisition; but, like a splendidly built bank *minus* an in-going capital, the brain is a poor concern without a good chest and stomach to feed it with its full compliment of oxygen, nitrogen, phosphorus, water, and other brain-bullion.

You have had enough with regard to the physical requirements of your pilgrimage. Now to the mental.

I need not urge upon you that, in the course of the true Medical Pilgrim, industry is the first element. You will remember what has been already said about force, fearlessness, and fact. I will not dwell therefore on this topic, but pass on to other considerations.

Let me forewarn you that, in these days, the science and practice of medicine are passing through a silent, constitutional, and withal a rapid revolution; a revolution so marked, that if in this half of the nineteenth century there is the same amount of progress that has characterised the previous half, the medicine of the twentieth century will scarcely contain a remnant of those principles and dogmas on which the medicine of the eighteenth century was based.

Are you astonished at such a statement? You need not be. The revolution is not peculiar to medicine, but to mind. It pertains to all sciences, to all the affairs of human kind. Step with me for a moment aside; give me your full thoughts, and we will seek for the proof of this theorem in one broad illustration.

Stripping ourselves for the time from this nineteenth century configuration, and unseen garment of mind in which we are enveiled, let some six centuries go by in panoramic dissolution, and let us pass with them into the century yet preceding.

We are British students still. Richard the Lion Hearted, our king, is away with his steel-plated knights to the Crusades, and we remain in the cloister, the science-men of the day. It is a brilliant night in this autumnal season; and we, the presumed interpreters of nature, the vesper song fainting on our ears, carry ourselves to some near mountain ridge, and discuss according to our knowledge and our dogmas the problems of the universe. Who is the wisest amongst us, is he that names the stars by their groups or constellations, and fixes a specially prophetic interpretation to the relative positions of those stars which never twinkle. We ask each other, What are stars? and the wisest of us says—"Lights merely to this one and great earth—the whole host but the illuminations of that great earth's ceiling." We speak of the sun as of its parading over this earth for daylight; and of the earth as of a great square of table-land resting on elephant's or tortoise's back. We turn to matter, and pronounce all that we cannot see, nothingness; laughing at those imbecile followers of Leucippus, who persist that matter is ultimately made up of particles that the eye can never reach. The wind wafts over our faces, and we call it the invisible element, the unknown moving force for the mere mariner's sail. We are the scholastic representatives of Richard's reign, and these are our dogmatical faiths. Let no heretic dispute them!

Back from the depths of history again; off with the cloister robe; and fainter the vesper song. The war is in India, not Palestine; and Victoria occupies the throne of the Lion-Hearted. The autumnal night is the same, the stars are the same, the earth is the same, the wind is the same, and we are the same, in that we represent once more the science-men of our day. We will climb the same hill at the same hour, and resume our mutual cogitations. Now on this hill a science-temple has been built, and filled with strange things. But are the stars mere night-lamps now? We place an eye to the tube of Galileo; and lo! the twinkling stars are suns, and the prophetic stars are worlds—worlds like ours. We guage the blue deep beyond and beyond the stars we saw before; and firmament opens beyond firmament, until the illimitable so terrifies us, that instead of our recognising one great world here with stars above for its grand illumination, the world altogether sinks into the infinity of nothingness, and we—even lower. We discourse of the sun now, and call him the motionless; or give to him a grand theoretical circle, which the mind can scarce conceive. We speak of the earth as a ball, and send it whirring round the sun for its seasons, and turning on itself for its day and its night. We turn our thoughts to matter; and behold! the followers of Leucippus are the lords of the argument. We put an eye to another tube, and from the infinity of immensity, we plunge into the infinity of minuteness and divide the unseen, —new worlds in both extremes. The night-breeze wafts over our faces, and we claim it as a known friend. We give to it a local habitation and a name; we give to it weight; we measure its force; we split it into two portions, definite themselves and well known; and we converse on all those things with one voice as though they had never been doubted, as though they formed part and parcel of the natural knowledge of man.

I have given you these two pictures in striking contrast, that you from them may see the grand revolutions of science, and may learn, in regard to your own section of science the intrinsic folly of binding your faiths inviolably to any dogmas or opinions, saving those which you can read, and mark, and learn on the unrestrained and simple authority of nature.

You think, perchance, that from these reasonings I am persuading you from the study of books. Not at all. It is true I would warn you from what may be called traditional book-learning; for the traditional book-makers are the lions, and sloughs, and sleeping bowers in the Medical Pilgrim's path. But, as books written from the direct observation of nature are guides to the reading of nature from nature, I would by all means recommend such books to your attention, not to supplant the natural study, but to direct it.

You climb Ben Lomond with a man who shows you the way, and who points out the peculiarities of the mountain; but the mountain is seen by your own sense, and you learn it from itself. The guide might not be a man but a book; and the natural study might not be a mountain, but the dead body of a man; yet the positions of the guide and of the natural study are the same.

Do you ask of me—Have we the facilities for this natural course of study? I answer; yes. More facility than for any amount of book study that you can comprehend. You study anatomy. Have you not the veritable book of anatomy in the dead man and his parts? You study physiology. Are not plant and animal in your hand? You study chemistry. Is not the laboratory with its every modern convenience at your disposal? You study the mechanical art of surgery. Is not the dead subject your surgical possession? You study disease; and lo! every phase of disease is brought within four walls for your contemplation. Need I say—yes, I must say, that there is no excuse either in morals or in knowledge, for that student of medicine who in these days is content to take his knowledge of natural things from pastors or masters, when he might take it from the Great Master over all. It is the worship of idols, such work, in the presence of the God of the universe.

Say you that final examination is the ordeal you dread and prepare for? Say you that final examination at College and

Hall is not conducted from nature but from sheep-skin? Do you remind me of the grumblings of the son of a newly appointed old examiner, who, himself a noodle, mourned his hard fate that he had weekly to grind up his father in the bones? Well, I hear all you have to say, and I know its force; but these are my deductions from it: that if you *know* from the natural *thing,* no examiner mortal can pluck you from a reading or a picture; while, if you learn grinder-fashion from a picture or reading, any examiner who will, can pluck, and will deservedly pluck you, from the natural thing.

Thus it is, that I warn you to go always to the originals for your information, and both now and hereafter to be very suspicious about other authorities.

And while you are thus yourselves learners from the realities of science, take care, as you advance into intellectual manhood, not to mislead others by constituting yourselves the authorities of nature. Gentlemen, believe me, there is not a greater quack extant than he who claims to himself and sticks himself up to this distinction. There can be but one authority in science—nature. It is true, a man may discover a natural law, or fact, and make it known; but in the very acts of his discovery and its publication, he resigns all claim to authority, by calling, as he does, on every passer-by to see the fact for himself. Such a man is of the greatest of men; but he would as soon claim to himself the authorship of the natural fact itself, as of its mode of operation.

Your authorities, your *bonâ fide* authorities—your self-enunciating authorities—are men of a cast precisely the opposite to the true discoverer. I will tell you what they are; they are the members of a thorough-going Cockney family, who, walking to the foot of Ben Lomond, pump the guide with note book in hand, straightway return home satisfied with their intimate knowledge of the mountain, and write an authority-description of the prospect from the mountain summit. These are your authorities—they are all to be found in the broad and easy way, amongst the *young ladies,* the *toadies,* and the *old women*—a special great batch, I see, amongst the "young ladies." I beg you never for a moment to cross over to them, for some of them are very seductive—scientific harlots of the first petticoat and brocade.

I repeat, then, that the sciences change, and that medical science is changing specially fast. I repeat that you must follow that change; and now it is my duty to indicate the points of the way, and to explain how you must direct your steps, differently from your fathers.

It was the custom in the olden times, and this custom is not entirely expunged from medicine, to follow in all medical studies that method of induction which endeavours to arrive at general laws by the enumeration of all particulars. That is to say, taking a simple illustration, it was endeavoured, by collecting all the possible causes of some simple effect, say a disease such as small-pox, and by collecting the histories of a vast number of instances of such effect, to deduce the real cause, by selecting, out of the number of seeming causes, that as the true one which presented itself the greatest number of times, and the most prominently. This line of induction is almost worthless, as any thinking man may see; because, after collecting an immense number of facts, the apparent prominent cause might after all be but a coincidence, or a consequence. The cause thus assumed was, therefore, not a proven cause, but an assigned one.

It remained for the illustrious Lord Bacon to illustrate the feebleness of this principle of inquiry, and to introduce another inductive system which bears his name, and which is based on the assumption, that as in nature everything is uniform and stable, and as whatever has occurred in nature will occur again, under the same influence, and present the same phenomena, so is it proper, in an inquiry into causes, not to draw inferences from the enumeration of particulars, but to deduce universal principles from the observations of particular experimental, or even accidental observations. Thus, as you will see, Lord Bacon threw out altogether the enumeration principle and moved into the particular. But the induction of Bacon went even further; it taught that, instead of collecting so many causes and selecting one as the most prominent and therefore the real cause, it were more correct to consider whether each cause might not, with greater truth, be weeded out of the argument, rather than wedded to it. Thus he separated nature by proper rejections and exclusions; and by such exclusion or clearing away of impossibles, he proposed to arrive at last at that cause which, in the absence of all other causes, was sufficient to account for all the facts connected with the phenomena under consideration.

I hope I have made this clear; because I wish to impress on you the fact that the Baconian system of induction is the one which you must follow, if you would do any thing solid in the process of medical discovery. You must not say in regard to any phenomenon, whether physiological, pathological, or symptomatical, "Lo! this is the cause, or that is the cause," inasmuch as such or such an apparent cause is present; but you must accept even the cause most probable as *problematical*, until you have proved that as a cause it does not admit of exclusion, and that all other apparent causes do admit of exclusion as apart from, and unnecessary to the development of the phenomena before you.

If you have understood these observations, and if you will follow them out in your future career, you will save yourselves from one of the most natural and fatal errors common to men who loosely follow scientific pursuits. You will not, when you see the working of any natural law, say at once, "There is the reason of this law"; you will not endeavour to pick out reasons for the law and apply these hypothetically to explain the law; for this any stupid or cunning deviser can do. But you will look at the facts as phenomena before you; from the study of these you will descend to the discovery of the one cause of the phenomena, and you will look only for one cause, as knowing that there cannot in nature be two causes for one effect. In the modern revolution of medicine the change from the general to the particular mode of induction is a leading feature; and you must consequently learn and work by the Baconian or exclusive method, or your labours will be as puerile as they will be diffusive.

In olden times, the study and practice of medicine was based on the mere observation of diseases themselves: and he was thought the greatest man who could split up symptoms into diseases *per se*, in the most elaborate and verbal manner. Hence the origin of such a flux of nonsense as Cullen's Nosology. In present times, this refinement of division is cast off, and men are advancing by true induction, back from symptoms to causes, and to *few* causes. They are beginning to talk about the elements of disease, and to limit these elements to the smallest possible number. They are beginning to connect disease-symptoms with certain natural functions, and to observe that symptoms are but exaggerations or depressions of natural acts. They are going further—they are aiming at the discovery of natural functions, and are foreseeing that when the natural act is understood, its aberrations will also be understood as a necessary sequence.

You true pilgrims must also follow this progressive course, —follow it rigidly and persistently.

In olden time, the manifestations or signs of disease were accepted as originating in the body itself. The causes of diseases were internal. Past and once renowned practice-of-physic-books were full of nothing so much as practical proofs that this disease originated in the liver, that in the blood, and so on. There were great schisms, too, in medicine, on the questions whether diseases in general originated in the solids or in the fluids. Hence arose such terms as humoral pathology and pathology of solids. These absurdities even yet stick to medical language. But the true science of medicine in these days laughs at the old dogma. Now, falling back on Baconian principles, and tracing phenomena towards their essences, and excluding the coincidences, which were once considered essentials, we have traced the causes of diseases out of the body altogether, and have proved that some ninety-nine out of a hundred maladies are from the external world.

How this one grand step has revolutionised medicine; how it has led to the building up of the new and noble fabric of hygienic medicine; how it has erased some diseases altogether; how it has simplified the study of disease; how it has brought the study of medicine into closer communion with chemistry, natural history, natural philosophy, national history; how it will yet influence our destinies as a profession—are points which can only be understood by those who are acquainted with the past history of our science, and who, standing now as prophets with their heads turned backwards, are able to read off from the history of the past the events of the future. And yet for your hands there remains much to be done. The great problem of the external cause of disease has to be resolved into its ultimates. Special causes of special diseases have yet to be hunted down. Where burrow the poisons of scarlet fever, of small-pox, of typhus? They are out in the universe. Do you isolate but one of them, and show its annihilation, and your pilgrimage is immortal.

In olden times, the treatment of diseases was based on the hypothesis of the internal or corporeal origin of the disease itself. The disease was located in the solids; therefore the solids must be strengthened or resolved. The disease was located in the fluids; therefore must the fluids be attenuated or condensed. Thus the idea of externals in the treatment of disease was excused altogether, and treatment was made up of one grand hypothetical system of modifying the condition of the body, or parts of the body, by medicaments, blood-lettings, scarifications, and the like. A great abuse thus crept into medicine; and such unquestionable evil resulted, that the acute observer Addison was led to utter the satire, "that doctors were divided into two classes,—those who slew in chariots, and those who slew on foot." The satire was overstrained, yet not wholly devoid of truth. Men locked up with fever in the foul atmospheres of jails, were left in such foul atmospheres, and were bled, purged, and blistered, to be—shall I say it?—*cured*. And errors of this kind, while almost universal, were the more glaring, because the very attempts at "cure" were based on the ignorance of empiricism.

In these days, the fact of the external origin of diseases has led some men into the opposite extreme, and the treatment of disease is left to the application of external means. Pure air is now the remedy for the typhus stricken, for the consumptive, and various other sufferers; and the natural process of cure is talked of as *the* cure, and the only cure. With regard to this modern phase in medicine, I would advise you cautiously, and by the laws of common sense. I would inform you that there are many diseases which, having a purely external origin, are removable by the withdrawal of their efficient cause, and are not removable by any narrower or less philosophical process. I would tell you, too, that many diseases which spring from the action of a poison taken into the body, get well by a kind of natural process, from the circumstance that the poison, being taken in as though it were a food, is modified as it passes through the animal laboratory, and being thrown out of the body by the excretions, is so lost that the removal of the symptoms, which were dependent on the presence of the poison, is ensured. I tell you this boldly; but I would not tell you that such natural elimination of the poison is a certain preservative from death, and that there is, therefore, an absolute *vis medicatrix naturæ*, or curative entity inherent in man. Not at all; for were this the case, all patients would naturally get well, which is not the fact. The truth lies in this,—that while in the natural or spontaneous elimination of the exciting causes of disease, cure is, in many cases, promoted, in other cases the very act of elimination leads to the setting up of such new and important physiological modifications that death is the result.

I leave it, therefore, on your minds, that you have three things to learn in the treatment of disease according to our modern knowledge: *first*, how to remove external causes; *secondly*, how far to trust to the spontaneous evolution or removal of causes which have taken effect on the body; and *thirdly*, how to meet those extreme results which sometimes succeed the natural or spontaneous attempts at removal.

In olden times, the science and art of surgery was a rude, and even cruel practice. "Spare not," was the surgeon's pass-word; and brilliancy with the knife was as sure a guide to worldly fame, as skilfulness with the sword. The surgeon not only supplemented nature, but dubbed her quack, and took her business from her ruthlessly. Wounds to be "cured" were filled with unguents. Limbs deformed were put out of sight three fathoms deep, or into sight in the dissector's museum.

In the modern revolution these brilliant exploits are no more. The sound flesh is simply left in natural apposition. The deformity is removed, while the limb still supports its owner; and Nature, instead of being snubbed, is permitted to stand on the card of all intelligent Sawbones as the senior partner in their concern.

You must not be behind in this progress; you must take your partner likewise, and give the lead.

The last series of these instructions brings before you the philosophical study of the action of medicines,—and here you have a great deal to learn; for, while you have not only to ascertain the action of medicaments on the body, you have to isolate the results produced by medicaments from those which would follow naturally, were no medicaments used at all. Here, indeed, is a pivot on which much important matter turns. Go into the actual practice of medicine, and you will see what I mean. Did you ever meet with a practitioner who did not consider himself the most successful of all practitioners? Did you ever meet with two practitioners who practised strictly alike? If, then, all are successful after a fashion—and I believe in this respect we are each of us much of a muchness; and if the line of practice pursued by each of us is not of an equality—*ergo,* our success is dependent on something more than our special modes of treatment; it is dependent on external or internal natural results, which we universally do not see or comprehend.

With you, the first-day Pilgrims in the narrow path of medicine, it rests to clear up these difficulties and differences, to distinguish the natural from the artificial means of cure, to unite the natural and the artificial means of cure; to prove how diseases may be prevented wholesale, and to look upon this as a part of your professional duty; to prove also how diseases, when they occur by accident, are removable in detail, and to feel that neither the one nor the other of these offices is the least, or the greatest.

To sum up all these points of instruction, two sentences suffice.

Work from nature; she is your only authority.

Work by induction; that is your only charter.

If on the mind of but one student here to-day I could imprint these simple lessons, I know not what benefit I may have bestowed on him, on his profession, on the world. All the good things which lie in store for medicine, are, indeed, embodied in the progress of medical philosophy.

You young men, going forth into medical life, ignore not, mistake not, where the citadel of your strength is founded. Be led away by no professional bigotry, by no professional intolerance. When you dictate, think how you can prove. When you blame the world for not recognising the full measure of your services, think how you can convince the world that your services are worth what you presume. When you abuse the quack for his impudence and shallow conceit, call to mind that the quack flourishes on your weakness. Recall the truth, that in astronomy there are no quacks; that in mathematics there are no quacks; that even in simple mechanics, even amongst the artisans who will one day form the lower strata of your patients, there are no quacks. And why? Because the men of these occupations, great or little, work to a demonstration, and are paid or penaltied according to their competency.

Pilgrims of this day, entering on your work, braving all difficulties, to you is left the positive demonstration of medical science: nothing less; nothing more. I would warn you from all by-paths,—political, servile, selfish,—that you may toil on in the narrow way. Colleges may dispense to you legal rights, and universities honours; but on these have no reliance, if such reliance interfere for a moment with your own self-trust. *You are the medicine, not the colleges.* Parliament *cannot* reform your profession, if you reform not your science. Parliament *need* not reform your charters of privilege, if you reform your charters of knowledge. What, if your success come not with the night? It will come with the morning; and will descend on you, not as a mere temporary success resting on thirty-six clauses of act of Parliament, and adapted to a few years of an ever-changing history, but on the permanency of truth and the solidity of nature. Work on in your difficult road, thinking not of the hour in which you live, but of the epoch which you were born to signalize. Work as for a scientific future even in this world. Work in this earnest and unimpassioned spirit; and that future may be so far secured that medical pilgrims, taking their first day's journey in years long to come, shall treasure up your existences with theirs. To this, the fullest reward of your pilgrimage, I direct your feet to-day; and, as you love yourselves, your profession, and your country, strive after it.

> "'Tis of more worth than kingdoms, far more precious
> Than all the crimson treasures of life's fountain:
> Oh! let it not elude your grasp; but, like
> The good old patriarch upon record,
> Hold the fleet angel fast, until he bless you."

